# Effects of primary angle-closure glaucoma on interhemispheric functional connectivity

**DOI:** 10.3389/fnins.2023.1053114

**Published:** 2023-02-09

**Authors:** Yongqiang Shu, Yuying Huang, Jingting Chen, Liting Chen, Guoqian Cai, Yu Guo, Shenghong Li, Junwei Gao, Xianjun Zeng

**Affiliations:** ^1^Department of Radiology, The First Affiliated Hospital of Nanchang University, Nanchang, China; ^2^Medical Imaging Center, The First Affiliated Hospital of Jinan University, Guangzhou, China

**Keywords:** glaucoma, functional magnetic resonance imaging, resting state, voxel-mirrored homotopic connectivity, functional connectivity

## Abstract

**Background:**

Previous studies on primary angle-closure glaucoma (PACG) primarily focused on local brain regions or global abnormal brain activity; however, the alteration of interhemispheric functional homotopy and its possible cause of brain-wide functional connectivity abnormalities have not been well-studied. Little is known about whether brain functional alteration could be used to differentiate from healthy controls (HCs) and its correlation with neurocognitive impairment.

**Methods:**

Forty patients with PACG and 40 age- and sex-matched healthy controls were recruited for this study; resting-state functional magnetic resonance imaging (rs-fMRI), and clinical data were collected. We used the voxel-mirrored homotopic connectivity (VMHC) method to explore between-group differences and selected brain regions with statistically significant differences as regions of interest for whole-brain functional connectivity analysis. Partial correlation was used to evaluate the association between abnormal VMHC values in significantly different regions and clinical parameters, with with age and sex as covariates. Finally, the support vector machine (SVM) model was performed in classification prediction of PACG.

**Results:**

Compared with healthy controls, patients with PACG exhibited significantly decreased VMHC values in the lingual gyrus, insula, cuneus, and pre- and post-central gyri; no regions exhibited increased VMHC values. Subsequent functional connectivity analysis revealed extensive functional changes in functional networks, particularly the default mode, salience, visual, and sensorimotor networks. The SVM model showed good performance in classification prediction of PACG, with an area under curve (AUC) of 0.85.

**Conclusion:**

Altered functional homotopy of the visual cortex, sensorimotor network, and insula may lead to impairment of visual function in PACG, suggesting that patients with PACG may have visual information interaction and integration dysfunction.

## 1. Introduction

Glaucoma is a trans-synaptic neurodegenerative disease characterized by optic atrophy and visual field defects. Moreover, it is also the second leading cause of blindness after cataracts, affecting about 80 million people worldwide (Pan and Varma, 2011). Neuropathological studies revealed that glaucoma is a trans-synaptic neurodegenerative disease involving the brain, and its pathological mechanism is similar to that of Alzheimer’s disease (Gupta and Yucel, 2007), indicating that it is not only an ophthalmological disease but also an encephalopathy. This means that it is important to understand their brain changes. On the other hand, clinical studies have confirmed that the visual acuity of some patients inevitably declines even after antihypertensive treatment, which confirms our theory from the opposite direction. In summary, the neuropathological mechanism of glaucoma still requires further exploration. Primary angle-closure glaucoma (PACG) occurs more frequently in Asian populations and is associated with a higher rate of blindness and heavier social burden compared with primary open-angle glaucoma (POAG), which is more common in European populations (Cheng et al., 2014). However, previous studies have focused primarily on the pathophysiology of POAG, whereas there is a scarcity of literature on PACG.

Resting-state functional MRI, a non-invasive technique for detecting brain function, has been widely used to investigate various neurodegenerative diseases involving the central nervous system. Our research group utilized rs-fMRI to explore spontaneous brain activity in patients with PACG with some success. Huang et al. (2015) found that PACG entails extensive visual cortex functional damage and visual cortex functional changes. On this basis, Jiang et al. (2019) confirmed that PACG exhibits different functional changes in the slow-4 and slow-5 frequency bands, suggesting that spontaneous neural activity in PACG is frequency-dependent. In addition, Cai et al. (2015) reported a compensatory increase in the DC values of the visual cortex in PACG after treatment, indicating that recovery of visual function after treatment may be attributed to functional reorganization in the visual cortex. Another study on functional connectivity density (FCD) revealed that patients with PACG exhibited different spatial distribution patterns in long- and short-range FCD (Chen et al., 2019). These studies reflect the characteristics and changes of the spontaneous activity of PACG from different perspectives. However, we made an often-overlooked observation: these abnormal brain activities were characterized by asymmetry and coupling between the bilateral hemispheres of the brain plays an important role in visual information processing (Terada et al., 2008). The mechanism and possible underlying significance of this lateral change in PACG remain unclear.

Mirror homotopy in brain function, that is, the high synchronization of spontaneous neuronal activity between symmetrical voxels in the bilateral cerebral hemispheres, is a fundamental feature inherent in the brain (Zuo et al., 2010). It represents the coordination and synchronization of functional activities between hemispheres and plays a vital role in visuospatial attention and executive function (Gracia-Tabuenca et al., 2018). Hence, Zuo et al. (2010) proposed the voxel-mirrored homotopic connectivity (VMHC) method that is primarily used to quantify the correlation between the time series of symmetrical voxels in the bilateral cerebral hemispheres, explore functional interactions between hemispheres, and reflect the process of information exchange and integration between hemispheres. VMHC has been used to explore the pathophysiological mechanisms underlying insomnia (Yan et al., 2018), monocular blindness (Shao et al., 2018), and obstructive sleep apnea (Liu et al., 2018). Unfortunately, there have been no studies on alterations in PACG interhemispheric functional connectivity.

Therefore, this study aimed to explore the changes in information interaction and integration between the hemispheres in PACG as follows: (1) VMHC was used to explore the homotopy in functional connectivity between the bilateral hemispheres of the brain. (2) Brain regions with significantly different VMHC values were used as seed points to explore changes in brain-wide functional connectivity. (3) Pearson’s correlation was used to analyze the relationship between VMHC values in abnormal brain regions and clinical scales. (4) A support vector machine (SVM) was used to determine whether VMHC and FC values could be used to distinguish PACGs from HCs. We hypothesized that multiple brain networks, including the visual network, are abnormally altered in PACG and that these abnormal functional alterations may be used to differentiate PACG from HC.

## 2. Materials and methods

### 2.1. Participants

This observational, case-control study was carried out in accordance with the Strengthening the Reporting of Observational studies in Epidemiology (STROBE) guidelines. The sample size calculation for this study was based on two-sample for testing the differences between patients with PACG and HCs. The power calculation was carried out on the online platform by NeuroPowerTools.^1^ Our study with an alpha error of 0.05 will require a total sample of 40 to test the association at 5% levels using a two-tailed test. From 2014 to 2017, a total of 40 patients with PACG and 40 age- and sex-matched healthy controls (HCs) were recruited from the First Affiliated Hospital of Nanchang University in Jiangxi Province, China for the study. All patients underwent appropriate ophthalmic examinations, including goniometric and tonometric IOP measurement, fundus optical coherence tomography, and visual field testing.

Inclusion criteria for patients with PACG were as follows: (i) typical glaucoma symptoms, such as tubular and central field of vision; (ii) monocular or binocular pressure > 20 mmHg; (iii) monocular or bilateral angle-closure according to gonioscopy; (iv) specific optic disc damage changes (discoloration or cup change); (v) right-handed; and (vi) aged between 20–70 years. Inclusion criteria for HCs were as follows: (i) no typical glaucoma symptoms; (ii) no other ocular disease; (iii) a naked eye corrected visual acuity (VA) of 1.0; (iv) right-handed; and (v) aged between 20–70 years.

The exclusion criteria for patients with PACG and HCs were as follows: (i) previously treated glaucoma; (ii) other eye diseases; (iii) psychiatric or other chronic underlying diseases; and (iv) brain parenchymal lesions.

This study was conducted in accordance with the Declaration of Helsinki and approved by the Medical Ethics Committee of the First Affiliated Hospital of Nanchang University. All participants provided written informed consent for the study.

### 2.2. Data acquisition

A Siemens Tesla 3.0 T MRI system (Siemens Medical Solutions, Erlangen, Germany) was used at the First Affiliated Hospital of Nanchang University, Nanchang city, Jiangxi Province, China. Participants were required to be in a neutral position during the scan, with sponges placed on either side to reduce head movement. Participants were instructed to keep their eyes closed and to remain relaxed, awake, and not thinking.

The resting-state fMRI (rs-fMRI) data were obtained using the following parameters: repetition time = 2,000 ms, echo time = 40 ms, flip angle = 90°, slice thickness/gap = 4.0/1 mm, field of view = 240 mm × 240 mm, in-plane resolution = 64 × 64, with 30 axial slices covering the entire brain, and 240 volumes acquired in 8 min. In addition, we obtained high-resolution brain structural images for each participant using a T1-weighted 3D MP-RAGE sequence (repetition time = 1 900 ms, echo time = 2.26 ms, flip angle = 9°, matrix = 256 × 256, FOV = 240 mm × 240 mm, thickness = 1.0 mm, and 176 sagittal slices).

#### 2.3.1. Data pre-processing

Data processing was performed using the MATLAB toolbox DPARSF6.0 (Data Processing Assistant for Resting-State fMRI^2^), which is based on SPM12^3^ as follows: (i) after format conversion, the first ten time points for each subject were discarded; (ii) temporal slice correction was performed on the remaining 230 volumes; (iii) head motion correction was performed to reduce small head movements during scanning, and participants moving more than 1.5° in any cardinal direction (x, y, z) were excluded; (iv) the individual high-resolution T1-weighted images were segmented using the DARTEL (Diffeomorphic Anatomical Registration Through Exponentiated Lie Algebra) tool and registered to form a new brain template; (v) the corrected functional images were registered into the Montreal Neurological Institute (MNI) space by the DARTEL tool and resampled at a resolution of 3 × 3 × 3 mm^3^; (vi) the CSF and ventricle signals were regressed to reduce the influence of confounding factors; (vii) the linear trend of the time course was removed, and then a band-pass performed temporal filtering (0.01–0.08 Hz).

#### 2.3.2. Voxel-mirrored homotopic connectivity

Pre-processed images were registered to a study-specific symmetric MNI template, and VMHC was calculated sequentially as the Pearson’s correlation coefficient between the residual time series of each pair of symmetric hemispheric voxels in the symmetrical MNI brain space. Subsequently, Fisher z-transformation was performed to improve the normality. Considering the influence of smoothing on the functional connection between hemispheres, we adopt the spatial smoothing of 0, 4, 6, and 8 mm full-width half-maximum Gaussian kernels, respectively.

#### 2.3.3. Seed-based functional connectivity

The brain areas that exhibited significantly different VMHC values between patients with PACG and HCs were set as regions of interest (ROIs). Functional connections associated with the ROIs and the remaining areas of the brain were calculated. Fisher’s z-transform was used to improve normality.

#### 2.3.4. Support vector machine (SVM) analysis

Based on Anatomical Automatic Labeling, we divided the whole brain into 116 regions, and then the VMHC and FC values of each brain region were extracted and used as features for machine learning analysis. To prevent the occurrence of dimensional disaster, the LASSO method was used to reduce the data dimension based on Python, leaving a total of 40 features. Then, a linear kernel-based SVM using the leave-one-out cross-validation method was used for classification evaluation. Accuracy, sensitivity, and specificity were calculated to quantify the cross-validation prediction performance of the SVM classifier, and the receiver operating characteristic (ROC) curve and area under the curve (AUC) were used to evaluate the model’s ability to discriminate.

### 2.4. Statistical analysis

Independent two-sample *t*-tests were performed to examine the general clinical variables using SPSS 26.0 (SPSS Inc., Chicago, IL, USA). Differences in age, handedness, ophthalmic examination, and sex between patients with PACG pre-treatment and HCs were examined using the Student’s *t*-test. The significance level was set at *p* < 0.05.

Voxel-based comparisons of all VMHC maps and secondary functional connections were performed using DPABI6.0. The two-sample *t*-test model was used, with framewise displacement as a covariate. All results were reported at the significant voxel level of *p* < 0.005, cluster > 26 voxels, AlphaSim corrected.

Furthermore, partial correlation was performed for PACGs to assess the correlations between the clinical variables [disease duration, IOP, visual acuity (VA), mean deviation of visual field (MDVF), horizontal cup-to-disc ratio (HCDR), vertical cup-to-disc ratio (VCDR), and retina nerve fiber layer thickness (RNFLT)]. Sex and age were regarded as covariates. The significance level was set at *p* < 0.05.

## 3. Results

### 3.1. Demographic and clinical features

The demographic and clinical characteristics of PACGs and HCs are shown in Table 1. As expected, compared with HCs, PACGs showed significantly higher IOP, HCDR, VCDR, and lower RNFLT, VA (*p* < 0.001). There were no significant differences in age and sex between patients with PACG and HCs (*P* > 0.05).

**TABLE 1:** Demographic characteristics in primary angle-closure glaucoma (PACG) subjects and healthy control (HC).

Category	PACG (*n* = 40)	HC (*n* = 40)	*P*
Age (year)	53.93 ± 11.15	52.73 ± 7.70	0.577
Sex (male/female)	24/16	26/14	0.11
Scik eyes (one/two)	12/28	N/A	
Disease duration (years)	1.41 ± 2.18	N/A	
Left IOP (mmHg)	26.11 ± 13.10	12.72 ± 2.16	<0.001
Right IOP (mmHg)	29.92 ± 13.82	12.92 ± 1.89	<0.001
Left VA	0.56 ± 0.42	0.90 ± 0.13	<0.001
Right VA	0.48 ± 0.35	0.97 ± 0.14	<0.001
MDVF Left (dB)	−12.32 ± 6.74	−1.1 ± 0.41	<0.001
MDVF Right (dB)	−12.45 ± 6.05	−1.0 ± 0.33	<0.001
Left HCDR	0.59 ± 0.22	0.35 ± 0.03	<0.001
Right HCDR	0.62 ± 0.23	0.34 ± 0.03	<0.001
Left VCDR	0.55 ± 0.23	0.34 ± 0.02	<0.001
Right VCDR	0.60 ± 0.24	0.35 ± 0.02	<0.001
Left RNFLT (μm)	90.48 ± 23.51	113.73 ± 4.54	<0.001
Right RNFLT (μm)	85.65 ± 23.93	112.70 ± 4.45	<0.001

IOP, intraocular pressure; VA, visual acuity; MDVF, mean deviation of visual field; HCDR, horizontal cup-to-disc ratio; VCDR, vertical cup-to-disc ratio; RNFL, retina nerve fiber layer; N/A, not applicable. <CPS

### 3.2. VMHC differences among groups and correlational analysis

**FIGURE 1:**
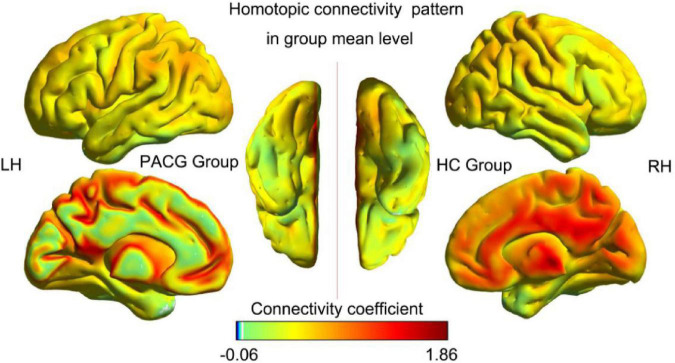
Voxel-mirrored homotopic connectivity (VMHC) spatial patterns at the group mean level of the primary angle-closure glaucoma (PACG) and healthy controls (HC) groups. LH, left hemisphere; RH, right hemisphere; HC, healthy control; PACG, primary angle-closure glaucoma.

**FIGURE 2:**
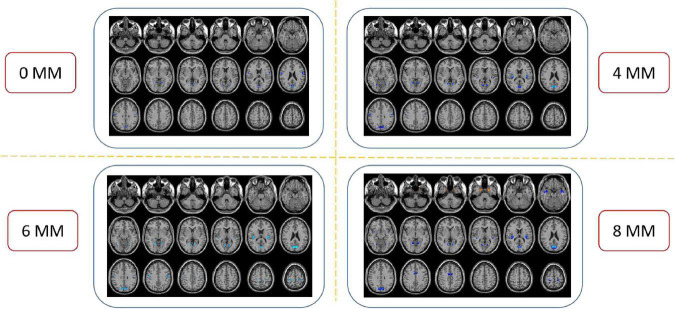
Compared to healthy controls (HCs), the patients with primary angle-closure glaucoma (PACG) showed remarkably similar altered voxel-mirrored homotopic connectivity (VMHC) brain areas at different level of spatial smoothing. FWHM = 0, 4, 6, and 8 mm. (*P* < 0.005, cluster > 26 voxels, AlphaSim corrected). The hot (cool) color indicates significantly increased (decreased) VMHC brain area. HCs, healthy controls; PACG, primary angle-closure glaucoma; VMHC, voxel mirrored homotopic connectivity.

**FIGURE 3:**
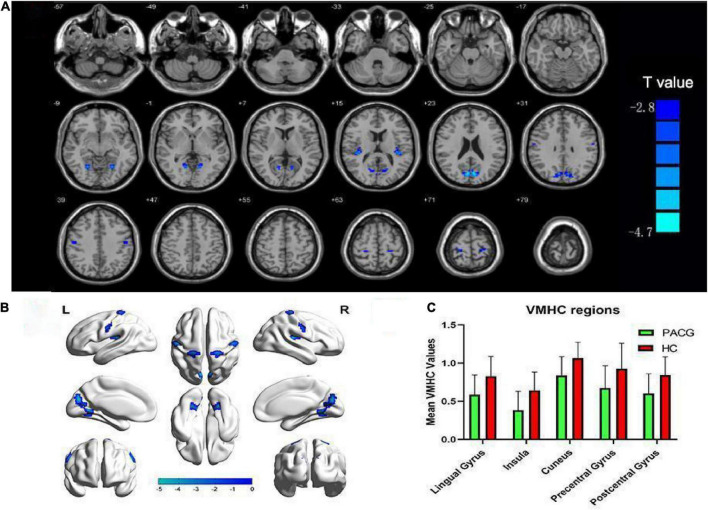
Altered voxel mirrored homotopic connectivity (VMHC) in patients with primary angle-closure glaucoma (PACG) versus healthy controls (HC). **(A,B)** Significant VMHC values were observed in the lingual gyrus, insula, cuneus, and pre- and post-central gyri; (*P* < 0.005; cluster > 26 voxels, AlphaSim corrected). **(C)** Mean values of altered VMHC values between the two groups. VMHC, voxel mirrored homotopic connectivity; HC, healthy control; PACG, primary angle-closure glaucoma.

The spatial pattern of VMHC at the average level of PACG and HC groups is shown as in Figure 1. Considering that the bilateral hemisphere VMHC of each group is symmetric, we have only shown the left hemisphere of patients with PACG and the right hemisphere of HCs. The stability analysis results are shown in Figure 2. As shown in the figure, except for the 0 mm group (no smoothing group), the differences between groups of 4, 6, and 8 mm are roughly similar, suggesting that it has good stability. The result of the 6 mm group was taken as the main result for subsequent analysis. Compared with HCs, patients with PACG exhibited decreased VMHC values in the bilateral lingual gyrus, insula, cuneus, pre-central gyrus, and post-central gyrus; no increased regions were detected between the two groups (Table 2 and Figure 3). However, No significant correlation was found between clinical characteristics and the abnormal VMHC in PACG group (*P* > 0.05).

**TABLE 2:** Differences in voxel-mirrored homotopic connectivity (VMHC) between two group.

Brain areas	MNI coordinates			BA	Voxels	*T* value
	X	Y	Z			
Lingual Gyrus	±24	−60	−9	19	74	−4.336
Insula	±42	−30	15	–	39	−4.320
Cuneus	±6	−78	24	18	73	−5.110
Pre-central Gyrus	±51	−12	36	–	27	−4.136
Post-central Gyrus	±21	−33	69	–	40	−3.522

VMHC, voxel mirrored homotopic connectivity; BA, Brodmann’s area; (x, y, z), coordinate of peak in the MNI space; *T* value, statistical value of peak voxel. AlphaSim corrected, *p* < 0.005, cluster size > 26.

### 3.3. Functional connectivity differences between groups

**FIGURE 4:**
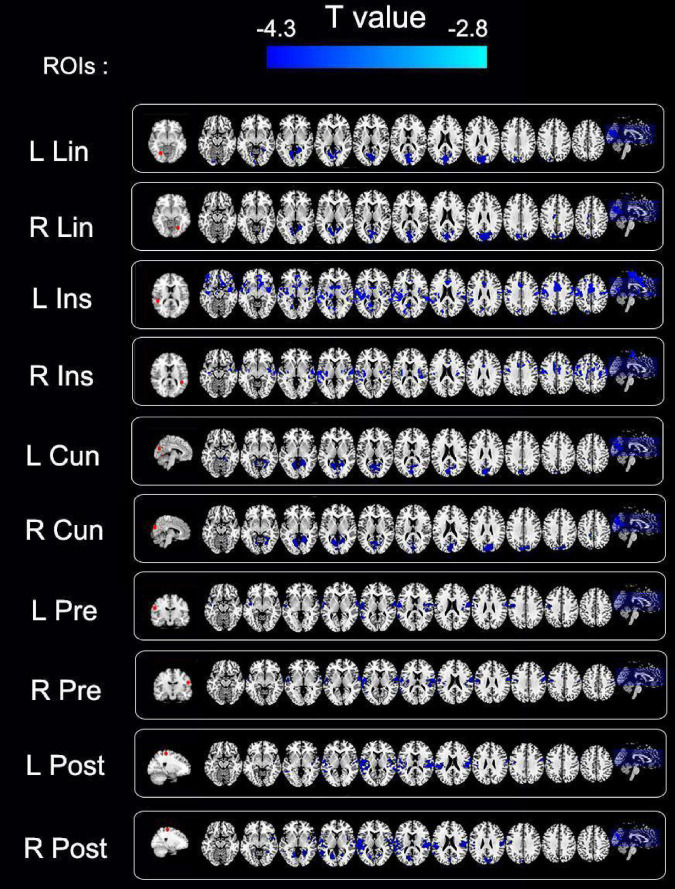
Group differences in functional connectivity of regions of interest (ROIs) between primary angle-closure glaucoma (PACG) and healthy control (HC). Significant functional connectivity values were observed in the default, salience, visual, and sensorimotor networks. ROIs, regions of interest; R, right; L, Left; Lin, lingual gyrus; Ins, insula; Cun, cuneus; Pre, pre-central gyrus; Post, post-central gyrus; cluster-level *P* < 0.005; cluster > 26 voxels, AlphaSim corrected.

The entire brain functional connectivity associated with five pairs of ROIs, in which the brain areas exhibited different VMHC values in the two groups, was examined. Significant changes in functional connectivity were detected in several regions of the brain. Reduced functional connectivity mainly involved the default mode network (DMN), salience network (SN), visual network (VN), and sensorimotor network (SMN) (Table 3 and Figure 4).

**TABLE 3:** Group differences in functional connectivity of regions of interest (ROIs) between primary angle-closure glaucoma (PACG) and healthy control (HC).

ROIs	Regions	MNI coordinates			voxels	*T* value
		X	Y	Z		
R Lingual	B Cuneus (62%)	−12	−63	0	1,168	4.315
	Precuneus (23%)					
	Posterior Cingulate (15%)					
	L Post-central Gyrus	−18	−51	60	108	4.435
L Lingual	B Cuneus (42%)	−18	−54	0	1,096	4.424
	Lingual Gyrus (19%)					
	Precuneus (28%)					
	Posterior Cingulate (11%)					
	L Post-central Gyrus (62%)	−9	−12	39	610	-4.127
	Pre-central Gyrus (37%)					
R Insula	R Anterior Cingulate (52%)	9	6	6	593	4.622
	Medial Frontal Gyrus (47%)					
	B Pre-central Gyrus (45%)	−3	3	42	4,697	5.113
	Post-central Gyrus (30%)					
	L Superior Temporal Gyrus (25%)					
	L Precuneus (37%)	−21	−78	18	411	4.815
	Posterior Cingulate (34%)					
	Cuneus (29%)					
L Insula	R Superior Temporal Gyrus (53%)	45	−6	12	419	4.42
	Insula (31%)					
	Middle Temporal Gyrus (16%)					
	L Superior Temporal Gyrus (62%)	−42	−36	6	465	4.89
	Middle Temporal Gyrus (38%)					
	B Pre-central Gyrus (49%)	39	−15	69	2,337	4.758
	L Post-central Gyrus (29%)					
	Medial Frontal Gyrus (22%)					
R Cuneus	B Cuneus (49%)	−15	−63	0	1,203	4.924
	Precuneus (22%)					
	Posterior Cingulate (29%)					
	L Post-central Gyrus (62%)	−30	−30	69	166	-3.512
	Pre-central Gyrus (38%)					
L Cuneus	B Cuneus (44%)	0	−84	27	1,301	4.699
	Lingual Gyrus (20%)					
	Precuneus (20%)					
	Posterior Cingulate (16%)					
	L Post-central Gyrus (44%)	−12	−42	51	342	4.015
	Precuneus (30%)					
	Pre-central Gyrus (26%)					
R Pre-central	L Pre-central Gyrus (52%)	−60	−3	12	472	4.763
	Superior Temporal Gyrus (28%)					
	Post-central Gyrus (20%)					
	R Pre-central Gyrus (48%)	63	−9	24	366	-4.314
	Insula (28%)					
	Post-central Gyrus (24%)					
	L Superior Temporal Gyrus	−63	−51	12	73	-3.757
L Pre-central	L Pre-central Gyrus (39%)	−60	−6	12	569	5.25
	Superior Temporal Gyrus (37%)					
	Post-central Gyrus (14%)					
	Insula (10%)					
	R Pre-central Gyrus (52%)	54	−6	−12	580	4.688
	Post-central Gyrus (18%)					
	Insula (15%)					
	Superior Temporal Gyrus (15%)					
R Post-central	R Insula	42	−18	−3	19	3.215
	R Pre-central Gyrus (29%)	51	−24	18	538	4.332
	Insula (27%)					
	Post-central Gyrus (24%)					
	Superior Temporal Gyrus (20%)					
	L Pre-central Gyrus (43%)	−48	−30	21	716	4.41
	Superior Temporal Gyrus (20%)					
	Post-central Gyrus (15%)					
	Insula (12%)					
	Inferior Parietal Lobule (10%)					
L Post-central	R Post-central Gyrus (30%)	51	−24	18	590	-4.438
	Superior Temporal Gyrus (30%)					
	Insula (24%)					
	Pre-central Gyrus (16%)					
	L Cuneus (58%)	−15	−54	−6	450	-4.3065
	Lingual Gyrus (54%)					
	Precuneus (18%)					
	R Parahippocampa Gyrus	33	−48	−6	196	-4.1728
	L Superior Temporal Gyrus (55%)	−45	−33	9	861	-4.5953
	Pre-central Gyrus (16%)					
	Post-central Gyrus (15%)					
	Middle Temporal Gyrus (14%)					

ROIs, regions of interest; BA, Brodmann’s area; (x, y, z), coordinate of peak in the MNI space; *T* value, statistical value of peak voxel; R, right; L, left. AlphaSim corrected, *p* < 0.005, cluster size > 26.

### 3.4. SVM prediction results

**FIGURE 5:**
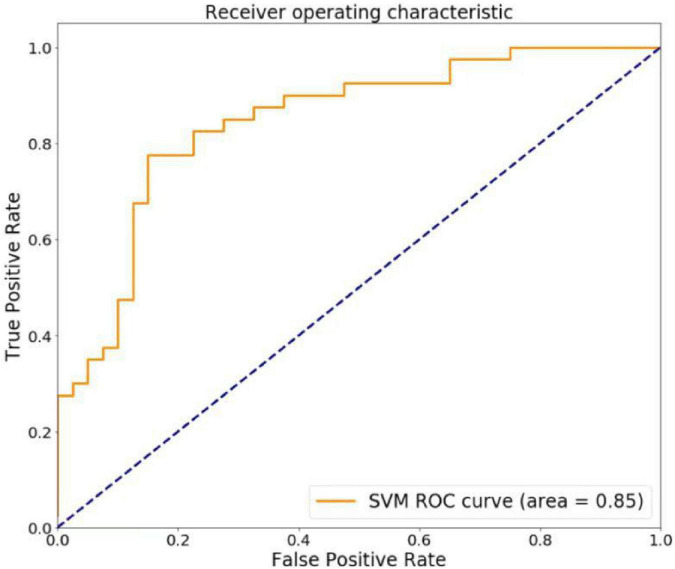
The regions of interest (ROC) curve of support vector machine (SVM) model. The SVM model showed good performance for the classification prediction of PACG, with an AUC of 0.85.

As shown in Figure 5, the classification prediction of SVM is conducted based on the VMHC and FC values. The SVM model showed good performance for the classification prediction of PACG, with an AUC of 0.85. The total accuracy, sensitivity, and specificity of the machine-learning classification were 0.80, 0.75, and 0.85, respectively (Figure 5).

## 4. Discussion

In this study, the VMHC method was used to explore changes in mirror homotopy in patients with PACG based on rs-fMRI technology. Our results show that, compared to HCs, patients with PACG exhibited reduced VMHC values in the bilateral lingual gyrus, insula, cuneus, and pre- and post-central gyri. The VMHC values in the insula were negatively correlated with the HCDR and VCDR of the left eye. Secondary seed-point functional connectivity analysis revealed decreased brain-wide functional connectivity in patients with PACG, mainly involving the DMN, SN, VN, and SMN. In addition, the SVM model showed good performance in the classification of adolescent depression by using different ReHo values as characters. In summary, these results suggest that visual dysfunction in patients with PACG may be attributable to functional changes in the visual cortex, which may be caused by an abnormal VMHC in the primary visual cortex. These findings shed light on new avenues and perspectives for PACG research and deepen our understanding of the neural mechanisms underlying PACG.

Compared with HCs, the VMHC values in the lingual gyrus and cuneus were lower in patients with PACG. Anatomically, both the cuneiform and lingual gyri are located in the primary visual cortex and are important components of the dorsal visual pathway, involving perception of spatial position (Frezzotti et al., 2016), processing (Wang et al., 2017), and encoding of visual information (Wang et al., 2016). In a study of strabismus and amblyopia, Liu et al. (2022) found that the functional connectivity values of the bilateral BA17 area and the left cuneiform lobe, the bilateral lingual gyrus. In addition, Cai et al. (2015) reported that the centrality of lingual and cuneiform networks was reduced in patients with PACG. To sum up, these findings suggest that functional impairment of the visual cortex in patients with ophthalmic diseases may cause visual dysfunction. Similar to previous studies, we observed decreased VMHC values in the lingual gyrus and cuneus in patients with PACG, which indicated a decrease in functional connectivity between the hemispheres, suggesting that information processing and coding in the bilateral primary visual cortex may be impaired.

In addition, decreased VMHC values in the pre- and post-central gyri were detected in our study. The pre-central (BA4) and post-central gyri constitute the main motor cortex (M1) and main somatosensory cortex (S1), respectively, and are key brain regions of the SMN. SMN play a crucial role in the control of eye movement (Iacoboni et al., 1997) and are involved in encoding of oculomotor activity (Blanke et al., 2000). Moreover, previous studies have shown that the SMN is closely related to spontaneous brain activity associated with the primary visual cortex (Wang et al., 2008), and the close connection between the two plays an important role in the processing of spatial visual information (Ringach et al., 1996). Jiang et al. used the regional homogeneity method to analyze patients with PACG, and the results revealed that the activities of the left pre-central and post-central gyri were reduced. Similarly, In a study of comitant exotropia, Zhu et al. (2018) observed decreased functional connectivity between the left visual cortex and left pre-central/post-central gyrus. Thus we indicate that after visual function impairment, the SMN is also affected to a certain extent by the reduction of visual signal input, suggesting that there may be abnormalities in the processing of spatial visual information in patients with PACG.

Moreover, decreased VMHC values were observed in the insula. The insula is believed to be associated with emotions, attention, and self-awareness (Kurth et al., 2010). At the same time, clinical studies have confirmed that patients with glaucoma often exhibit emotional cognitive abnormalities such as anxiety and depression (Kong et al., 2015). Thus, we speculate that decreased functional synchronization of the bilateral insula may reflect changes in PACG affecting cognition. On the contrary, the insula is a vital component of the orbitofrontal cortex and is closely associated with low spatial frequencies, subsequently transmitting coarse information about the visual scene (Chaumon et al., 2014). The orbitofrontal cortex plays a key role in facilitating visual input recognition by sending predictive feedback based on rapid low spatial frequency processing to the sensory cortex (Kveraga et al., 2007). Decreased VMHC values in the insula may also indicate that the transmission and initial recognition of visual scenes in patients are affected.

Finally, we used all regions of the brain exhibiting abnormal VMHC as seed points to conduct whole-brain functional connectivity analysis. Our results revealed that the main abnormal brain regions with reduced FC values were mainly involved in the DMN, SN, VN, and SMN, suggesting that there are extensive brain network changes in PACG. Firstly, Consistent with the previous description, we further confirmed that PACG is associated with functional loss in the VN and SMN, and anomalies of the SN and DMN were noted. The DMN is primarily a set of non-task activities that are paused during task activation (Puledda et al., 2021). Martin et al. showed that spatial visual information is encoded within the DMN through deactivation relative to baseline, which also means that the DMN may be a set of advanced visual areas (Szinte and Knapen, 2020). Secondly, the SN is closely related to the DMN, mainly by deactivating the DMN, promoting task-related information processing, and transmitting stimulus signals (Bressler and Menon, 2010). At last, the anatomical connection between the VN and SN has also been confirmed (Uddin et al., 2010). Hence, we can propose a reasonable hypothesis: the decreased interhemispheric information processing function of the VN in patients with PACG may lead to the functional changes in the SN receiving external stimuli, and moreover, the DMN, which is a high-level visual area, may also suffer damage.

At present, the diagnosis of PACG mainly relies on clinical symptoms and follow-up ophthalmological examinations, but some patients with atypical symptoms remain undiagnosed. Machine learning is an objective method that may lead to a higher diagnostic reliability for PACG. To evaluate efficiency of the SVM classifier, we also performed the ROC analysis. Based on the leave-one-out cross-validation technique, the overall recognition accuracy of the support vector machine was 80.00% and the AUC was 0.85. Therefore, abnormal signal values in these brain regions can be used as potential imaging markers to differentiate PACG patients from controls.

This study has some noteworthy limitations. First, the patients with PACG often experience different degrees of depression and other emotional changes, and the patients recruited in this study were not evaluated using emotional scales. The study therefore lacked relevant parameter guidance and failed to reflect the relationship between emotional changes and abnormal brain function. Second, although the research group recruited patients for follow-up after treatment for PACG, only 10 participants fulfilled the requirements, and the resultant sample size was insufficient to complete the comparison between pre- and post-operative follow-up. Recruiting more post-operative patients is required for further research. Third, the causes of abnormal VMHC have not been elucidated: whether it is caused by the abnormal spontaneous activity of the brain region itself or the abnormal connection of the fibers connecting the brain regions. This is an interesting focus for future research.

In conclusion, Our study showed that PACG individuals exhibited reduced VMHC values in lingual gyrus, insula, cuneus, and pre- and post-central gyri, while secondary FC showed abnormal networks including the default mode, salience, visual, and sensorimotor networks. These findings indicate that abnormal functional connectivity might be involved in the pathophysiological mechanisms of PACG.

## Data availability statement

The raw data supporting the conclusions of this article will be made available by the authors, without undue reservation.

## Ethics statement

The studies involving human participants were reviewed and approved by Medical Ethics Committee of The First Affiliated Hospital of Nanchang University. The patients/participants provided their written informed consent to participate in this study.

## Author contributions

XZ: conceptualization. GC: methodology. JC, YG, and JG: formal analysis. YS and YH: writing—original draft preparation. LC and SL: writing—review and editing. XZ: funding acquisition. All authors contributed to the article and approved the submitted version.

## Footnotes

1
http://www.neuropowertools.org/

2
http://www.restfmri.net

3
http://www.fil.ion.ucl.ac.uk
